# Analysis of retromuscular drain output and postoperative outcomes for heavyweight versus mediumweight polypropylene mesh following open ventral hernia repair

**DOI:** 10.1007/s10029-024-02972-7

**Published:** 2024-02-26

**Authors:** V. Essani, S. M. Maskal, R. C. Ellis, N. Messer, C. Tu, B. T. Miller, C. C. Petro, L. R. A. Beffa, D. M. Krpata, A. S. Prabhu, M. J. Rosen

**Affiliations:** 1 Case Western Reserve University School of Medicine, Cleveland, OH USA; 2 Department of General Surgery, Cleveland Clinic, 2049 E 100th St, Desk A-100, Cleveland, OH 44106 USA

**Keywords:** Abdominal wall reconstruction, Ventral hernia repair, Drain, Mediumweight, Heavyweight mesh

## Abstract

**Purpose:**

Heavyweight polypropylene (HWPP) mesh is thought to increase inflammatory response and delay tissue integration compared to mediumweight (MWPP). Reactive fluid volume (i.e., drain output) may be a reasonable surrogate for integration. We hypothesized that daily drain output is higher with HWPP compared to MWPP in open retromuscular ventral hernia repair (VHR).

**Methods:**

This is a post-hoc analysis of a multicenter, randomized clinical trial conducted March 2017–April 2019 comparing MWPP and HWPP for VHR. Retromuscular drain output in milliliters was measured at 24-h intervals up to postoperative day seven. Univariate analyses compared differences in daily drain output and time to drain removal. Multivariable analyses compared total drain output and wound morbidity within 30 days and hernia recurrence at 1 year.

**Results:**

288 patients were included; 140 (48.6%) HWPP and 148 (51.4%) MWPP. Daily drain output for days 1–3 was higher for HWPP vs. MWPP (total volume: 837.8 mL vs. 656.5 mL) (*p* < 0.001), but similar on days 4–7 (*p* > 0.05). Median drain removal time was 5 days for both groups. Total drain output was not predictive of 30-day wound morbidity (*p* > 0.05) or hernia recurrence at 1 year (OR 1, *p* = 0.29).

**Conclusion:**

While HWPP mesh initially had higher drain outputs, it rapidly returned to levels similar to MWPP by postoperative day three and there was no difference in clinical outcomes. We believe that drains placed around HWPP mesh can be managed similarly to MWPP mesh.

## Introduction

Polypropylene is a commonly used permanent synthetic mesh in open retromuscular ventral hernia repair. Mesh weight, or density, is one of the key technical specifications of polypropylene that surgeons should consider when selecting a hernia prosthesis. Historically, lightweight (LWPP) polypropylene (<40 g/m^2^) was designed to overcome perceived shortcomings of heavyweight (HWPP) polypropylene (>75 g/m^2^). Clinically, these purported advantages of lightweight mesh in terms of inflammatory response [1–4] and decreased pain [5] have been offset by increased hernia recurrence rates and a concerning incidence of central mesh fractures [6–8]. Mediumweight (MWPP) polypropylene (40–60 g/m^2^) was later introduced to balance the advantages and limitations of LWPP and HWPP. Mediumweight mesh has gained widespread acceptance as a suitable alternative to HWPP [9]. However, several recent retrospective analyses of large series of retromuscular hernia repair with mediumweight mesh suggest a mesh fracture rate of 4–5% [10, 11]. While there have been recurrences with HWPP, to our knowledge, no reported cases of mesh fracture have been documented with heavyweight polypropylene mesh. HWPP may offer the most stability without increased complications once tissue integration has occurred [12]. However, the concern of heavyweight polypropylene’s performance in open retromuscular hernia repair contributes to a general reluctance of many reconstructive surgeons to utilize HWPP mesh. One such concern is the less porous nature of the material and the potential for increased inflammatory response, delayed tissue integration and potentially more wound-related complications [1, 2].

Krpata et al. [13] recently reported the long-term outcomes of a randomized clinical trial evaluating the differences between HWPP and MWPP for open retromuscular ventral hernia repairs and found similar clinical and patient-reported outcomes at 1 year postoperatively. In spite of similar outcomes in the trial, surgeons at our institution continued to manage drains differently between HWPP and MWPP, citing a theoretical concern for slower tissue integration with HWPP. We performed a post-hoc analysis of this trial to evaluate clinical indicators of early mesh performance differences between HWPP and MWPP. Specifically, we suspected that increased inflammation and slow tissue integration may be expected to be seen as persistently high retromuscular drain output as well as differences in clinical mesh performance. We hypothesized that HWPP would exhibit higher retromuscular drain output as compared to MWPP.

## Methods

This is a post-hoc analysis of a multicenter, single-blinded, parallel-group randomized controlled trial, which compared heavyweight and mediumweight polypropylene (HWPP and MWPP, respectively) mesh for open retromuscular ventral hernia repair. The trial was registered on ClinicalTrials.gov (NCT03082391) and both the trial and this post-hoc analysis were approved by our institutional review board. Details of that trial have been previously reported; this current analysis was not specified a priori [13]. In brief, all patients were 18 years or older who underwent an elective, single stage, open retromuscular reconstruction of a clean (CDC wound class 1) midline abdominal wall defect width less than or equal to 20 cm measured intraoperatively. Patients included in this analysis underwent open retromuscular ventral hernia repair with either HWPP (>75 g/m^2^; Bard® Mesh (BD) or Prolene (Ethicon)) or MWPP (40–60 g/m^2^; Bard Soft Mesh (BD), Prolene Soft Mesh (BD), or Parietene (Covidien)) mesh at our institution between March 14, 2017, to April 17, 2019 and the primary outcome was pain at 1 year postoperatively as measured by the National Institutes of Health (NIH) Patient-Reported Outcomes Measurement Information System (PROMIS) Pain Intensity Short Form 3a. Patients were excluded from the trial if the fascial defect was >20 cm in width, primary fascial closure could not be obtained, or CDC wound class was >1. Institutional review board approval was granted at our institution for this analysis.

For the current analysis, a supplemental electronic medical record review was conducted to determine daily output from the retromuscular drains, in contact with the prosthetic, in milliliters (mL) for inpatient hospital stay up to 7 days postoperatively. The primary aim was to compare differences between postoperative retromuscular drain output between repairs with HWPP and MWPP mesh. Secondary outcomes included time from surgery to drain removal, hernia recurrence at 1 year, surgical site infection (SSI) within 30 days, surgical site occurrences (SSO) within 30 days, and surgical site occurrences regarding procedural intervention (SSOPI) within 30 days based on drain output. Hernia recurrence was defined using a pragmatic definition previously described by Krpata et al. [13] taking into consideration patient-reported bulge, clinical exam and radiographic evaluations. SSI, SSO, and SSOPI were defined according to the definition of Haskins et al. [14]. Drain management was left to surgeon discretion. The typical practice of surgeons at this institution is to remove drains when the output is <30–50 mL/day, but there is variation based on multiple factors including case complexity, wound class, discharge disposition, and mesh type.

### Statistical analysis

Data were described using median and interquartile range for continuous variables and counts and percentages for categorical variables. Longitudinal drain output between HWPP and MWPP groups was compared using a mixed effect linear regression model. Multiple logistic regression analysis was conducted to assess the associations between total drain output in the first seven postoperative days and 1-year recurrence, 30-day surgical site infection, 30-day surgical site occurrence, and 30-day surgical site occurrence requiring procedural intervention while adjusting for mesh weight, BMI, history of diabetes mellitus, and hernia width. Statistical significance was accepted as a *p*-value equaling 0.05 and R software package version 4.2.1 (The R Foundation, Vienna, Austria) was used for analysis.

## Results

During the trial, 288 patients were operated on at the Cleveland Clinic Center for Abdominal Core Health and met inclusion criteria; 140 (48.6%) received heavyweight mesh and 148 (51.4%) received mediumweight mesh. Patient demographics and baseline comorbid conditions were similar between the two groups (Table 1).

**Table 1:** Patient characteristics

Variable	HWPP *N* = 140	MWPP *N* = 148
Sex, *m*, *n* (%)	74 (53)	71 (48)
Age, *y*, average (SD)	59.97 (11)	59.09 (11.2)
BMI, kg/m^2^, average (SD)	32.1 (5.66)	31.9 (5.34)
Hernia Width, cm, average (SD)	13.8 (3.56)	14.26 (3.65)
Hypertension, *n* (%)	98 (70)	75 (51)
Diabetes, *n* (%)	34 (24)	34 (23)
COPD, *n* (%)	15 (11)	10 (7)
Smoking history, *n* (%)	1 (0)	7 (5)
Recurrent incisional hernia, *n* (%)	73 (52)	77 (52)
ASA Class, *n* (%)		
Class I	0 (0)	0 (0)
Class II	19 (14)	34 (23)
Class III	117 (84)	112 (76)
Class IV	4 (3)	2 (1)
Class V	0 (0)	0 (0)

*COPD* Chronic obstructive pulmonary disease

**Fig. 1:**
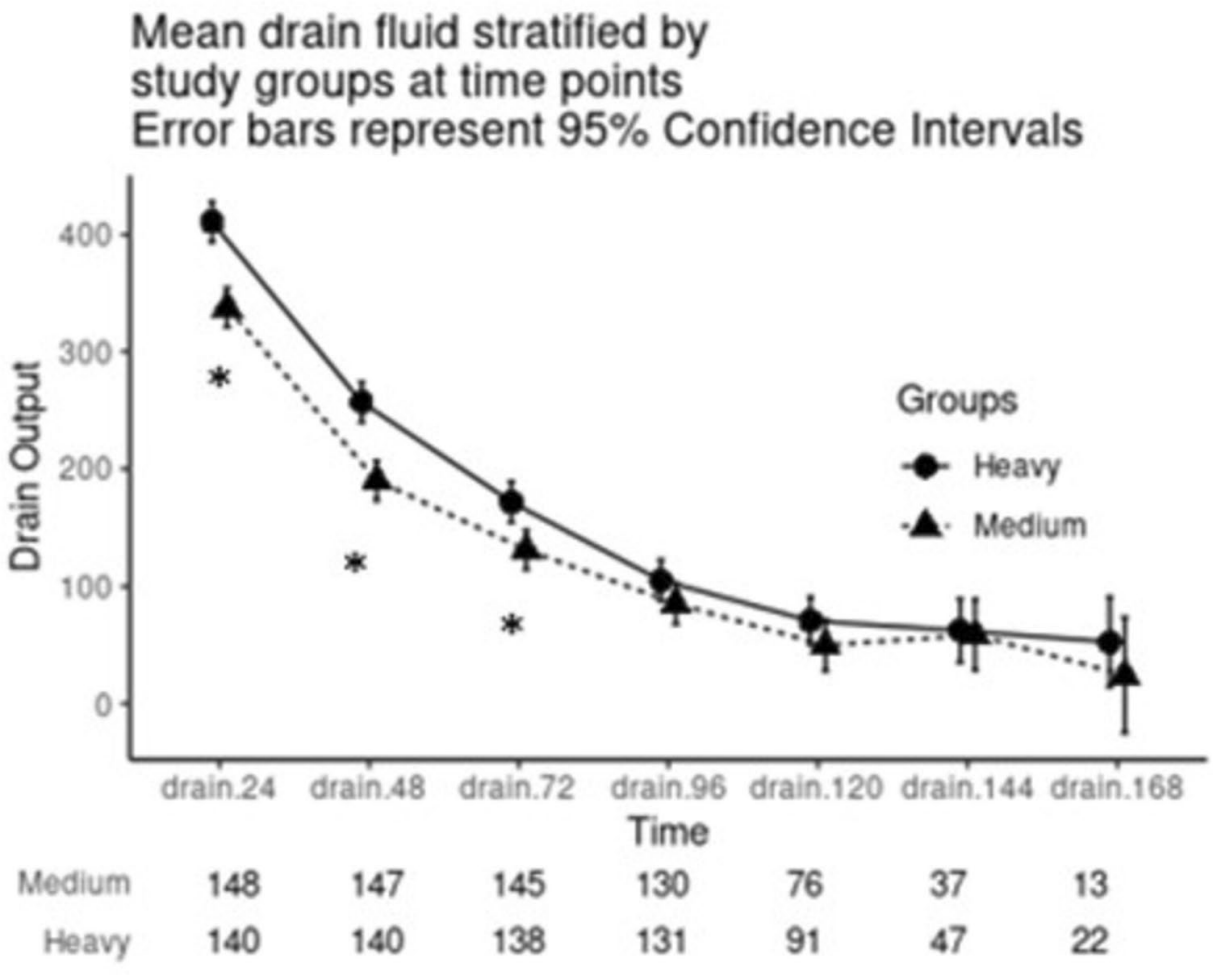
Median daily drain output over the first 7 days in 24-h intervals, stratified by HWPP and MWPP. Asterisk indicates *p*-value <0.05

**Fig. 2:**
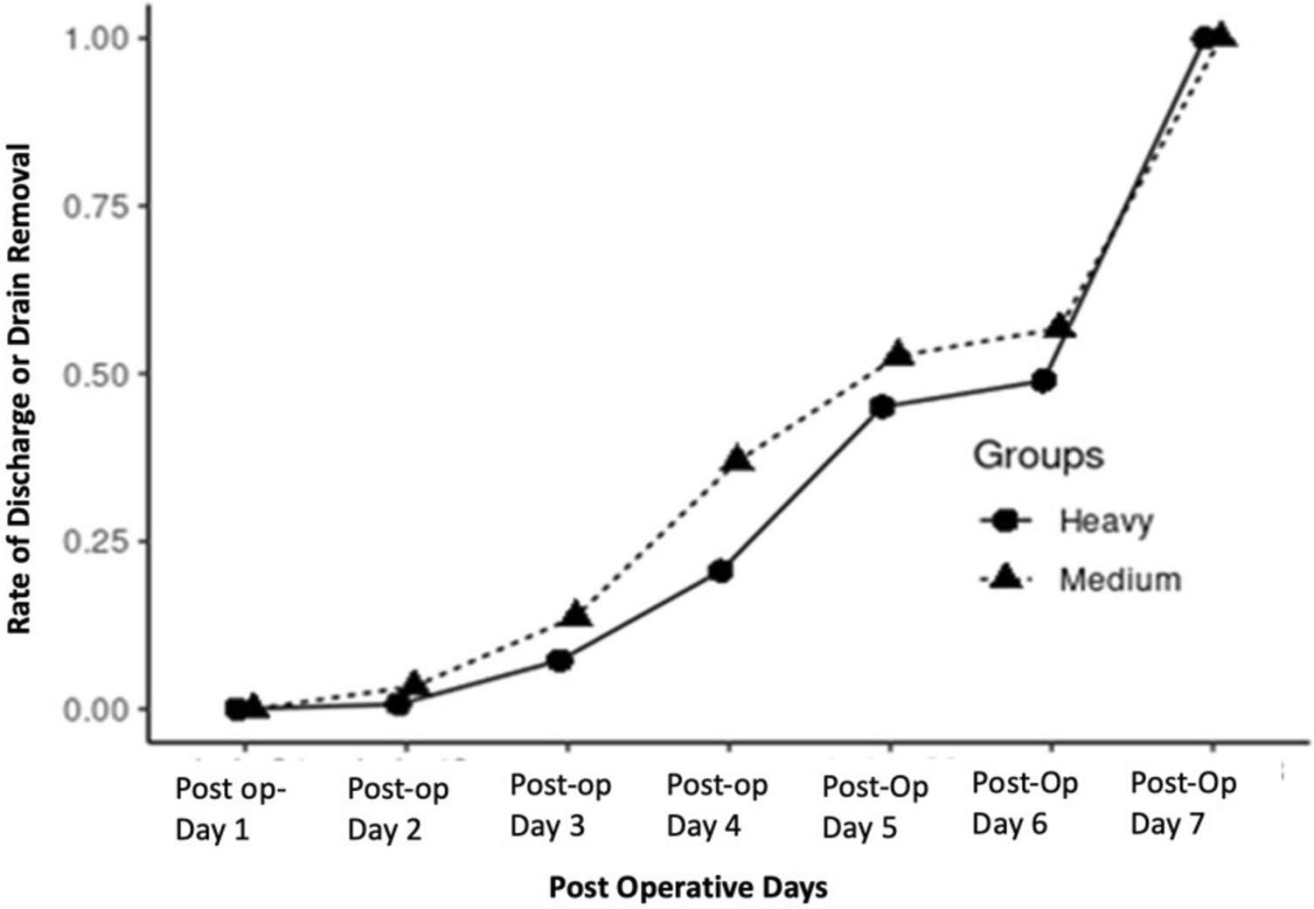
Time to drain removal or discharge, stratified by HWPP and MWPP

Daily drain output for the first 3 days was higher in the HWPP group as compared to the MWPP group (*p* < 0.001 for all), but drain output was similar after that (*p* > 0.1 for all) (Fig. 1). Median time to drain removal was 5 days for both HWPP (IQR, 4–6 days) and MWPP (IQR, 4–5.25 days) with no difference in rate of drain removal (*p* = 0.98, Fig. 2).

SSIs occurred at a rate of 4 (3%) in the HWPP group and 9 (6%) in the MWPP group. 21 SSOs occurred in the HWPP and 20 SSOs occurred in the MWPP group. SSOPIs occurred at a rate of 5 (4%) in the HWPP group and at a rate of 10 (7%) in the MWPP group (*p* > 0.05 for all). After adjusting for mesh weight, BMI, history of diabetes, and hernia width, total drain output in the first seven postoperative days was found to have no relationship to hernia recurrence at 1-year follow-up (OR 1, 95% CI 1.00–1.00, *p* = 0.29). Adjusting for the same variables, the total drain output was not predictive of SSI (OR 1, 95% CI 1.00–1.00, *p* = 0.11), SSO (OR 1, 95% CI 1.00–1.00, *p* = 0.5), or SSOPI (OR 1, 95% CI 1.00–1.00, *p* = 0.11).

## Discussion

This post-hoc analysis of a large randomized controlled trial is the first to compare differences in drain outputs after retromuscular ventral hernia repairs utilizing different mesh weights. We found a slightly higher initial drain output for the first 3 days postoperatively with heavyweight mesh; however, the drain outputs in both groups during this period were higher than our clinical threshold for removal. In addition, drain outputs decreased to <50 ml/day and were removed at a similar rate in both groups. We were further unable to correlate drain outputs with clinically meaningful outcomes. Given this information, it seems that heavyweight and mediumweight mesh materials perform comparably regarding drain outputs and clinical indicators of mesh performance, challenging our hypothesis that mesh integration rates would be slower in heavyweight polypropylene. While drain management varied between mesh weights, it appears that drains in both HWPP and MWPP repairs may be treated similarly with regards to timing for removal.

The clinical impact of mesh weight on surgical outcomes on hernia repairs remains debated, with many surgeons committing to a medium weight mesh due to historical concerns with both light and heavy weight mesh. There is strikingly little clinical data to suggest a difference in performance of mesh by weight. One of the reasons MWPP mesh was developed was to strike a balance between the potential advantages and disadvantages that exist in lightweight and heavyweight synthetic mesh. One of the commonly cited theoretical advantages of lighter weight mesh is that, compared to HWPP, it is comprised of less prosthetic material with larger pore sizes which theoretically generates less inflammatory reaction postoperatively leading to better tissue integration, but this has not been consistently demonstrated in animal models [15, 16]. Moreover, some clinical trials suggest worse outcomes with lighter weight materials versus heavyweight mesh in inguinal hernia repairs [17]. Our original randomized clinical trial further suggests that little clinical difference exists between mediumweight mesh and heavyweight mesh in retromuscular ventral hernia repairs [13]. Despite these data, many surgeons still believe that the smaller pores and heavier density material might impede mesh tissue integration. In this trial, we utilized drain output as a surrogate for mesh tissue integration. We hypothesized that as the material integrates into the surrounding tissue, the amount of drain output should decrease. While we did note drain output differences in the first few days these outputs were well above the threshold for drain removal and did not result in clinically significant differences in mesh performance or timing of drain removal.

The use of drain output as an indicator of mesh integration is a novel concept extrapolated from previous studies on the fluid mechanics and tissue reactions of different mesh materials. In an in vitro study of mesh permeability to fluid at different pressures, Jin et al. [18] demonstrated that the porosity of mesh materials and adhesive barriers impact fluid movement. Larger pore size mesh has been further correlated with improved integration, marked by increased neovascularization and fibrosis, as well as lower inflammation in multiple pig model studies [1]. Although the pathophysiology is not fully understood, fluid analysis of postsurgical seromas has demonstrated high levels of Th2/Th17 pathway cytokines [19], supporting a connection between inflammatory reaction and fluid accumulation. Taking these experimental models together, it could be deduced that denser, less porous mesh material would generate more foreign body reaction and fluid generation, while also demonstrating lower permeability to that fluid. That fluid would be seen clinically as drain output, which would decrease as the material integrates with the surrounding tissue. In the setting of ventral hernia repairs with transversus abdominis release, fluid accumulation would be expected based on the inflammatory response from dissection of the abdominal wall and creation of a large pocket within the retromuscular plane. The amount of fluid accumulation may vary with the specific mesh material [2] placed and its porosity which could be expected to translate clinically as higher surgical drain output. We acknowledge this hypothesis requires further basic science experimentation, however, we do believe that it is a novel potential opportunity to assess mesh integration clinically.

While the role of retromuscular surgical drains is debated [20–23], they are often placed to evacuate fluid from the potential space developed during a transversus abdominis release to promote tissue apposition with the mesh and improve integration [15]. Notably, in our study, average total drain output was between 300 and 400 mL/day (150 mL per drain) for the first day in each group. This is certainly a high amount of fluid and supports the clinical decision to place drains within this space. Importantly, our study did not include a control arm of non-drained patients, and as such, we are unable to establish a true benefit of drains. A recent meta-analysis found that drain placement at the time of retromuscular ventral hernia repair may also reduce the risk of developing a seroma (OR 0.34), though it did not decrease the risk of SSI, hematoma, SSO, or SSOPI [24]. While it is common practice to place retromuscular drains, the decision-making process surrounding surgical drain management is often based on theoretical assumptions about the significance of the volume of output. Indeed, there is a paucity of evidence and no consensus in the literature on when to remove postoperative drains [25]. Many surgeons use a set volume criterion for drain removal (i.e., 30–50 mL/day) and may keep the drain in place until their threshold is reached. However, often other factors are also considered when deciding drain removal management, including wound characteristics, mesh type, and patient functional status and social support.

Surgical drains can be an uncomfortable nuisance for patients and there is evidence that early drain removal does not increase infection or seroma/hematoma risk [26], while others cite the complications of drains including hemorrhage, inflammation, and infection [27]. Our data similarly suggest that the volume of output in the first 7 days has no clear association with short-term wound complications or early hernia recurrence. Leaving drains longer for HWPP mesh is unlikely to confer clinical benefit and based on our analysis can be more comfortably pulled prior to discharge. However, further prospective trials are needed to evaluate both drain utility and optimal duration of use.

There are multiple limitations of this study that deserve mention. As a post-hoc analysis, all outcomes are exploratory. We did not control surgeon preference for drain removal and although this is a single center trial, there might be some subtle variation in drain management per surgeon preference. Drain output and time to removal may be confounded by patient and operative factors which cannot be captured retrospectively. By capturing data for only the first 7 days, we also incompletely represent patients who were discharged with a drain in place for extended periods of time, although most surgeons preferentially remove drains prior to discharge. Finally, our choice to utilize drain output as a surrogate marker for mesh tissue integration could be inaccurate. While we feel, it is the best measure we have, more data would be required to show that this marker reliably measures tissue integration.

## Conclusion

While HWPP mesh had slightly higher outputs for the first 3 days, it rapidly returned to outputs similar to MWPP mesh. We believe that our findings support the concept that drains placed around HWPP mesh can be managed similarly to MWPP mesh. Finally, drain output volume in the first postoperative week should not be used as an independent predictor of short-term morbidity or long-term hernia recurrence.

## References

[1] Weyhe D, Cobb W, Lecuivre J, Alves A, Ladet S, Lomanto D, Bayon Y (2015). Large pore size and controlled mesh elongation are relevant predictors for mesh integration quality and low shrinkage—systematic analysis of key parameters of meshes in a novel minipig hernia model. Int J Surg.

[2] Klinge U, Klosterhalfen B, Müller M, Schumpelick V (1999). Foreign body reaction to meshes used for the repair of abdominal wall hernias. Eur J Surg.

[3] Bilsel Y, Abci I (2012). The search for ideal hernia repair; mesh materials and types. Int J Surg.

[4] Schmidbauer S, Ladurner R, Hallfeldt KK, Mussack T (2005). Heavy-weight versus low-weight polypropylene meshes for open sublay mesh repair of incisional hernia. Eur J Med Res.

[5] Moreno-Egea A, Carrillo-Alcaraz A, Soria-Aledo V (2013). Randomized clinical trial of laparoscopic hernia repair comparing titanium-coated lightweight mesh and medium-weight composite mesh. Surg Endosc.

[6] Petro CC (2015). Central failures of lightweight monofilament polyester mesh causing hernia recurrence: a cautionary note. Hernia.

[7] Blair LJ (2015). Lightweight vs midweight polypropylene mesh in 948 open ventral hernia repairs (OVHR). J Am Coll Surg.

[8] Žuvela M, Galun D, Djurić-Stefanović A, Palibrk I, Petrović M, Milićević M (2014). Central rupture and bulging of low-weight polypropylene mesh following recurrent incisional sublay hernioplasty. Hernia.

[9] Rosen MJ (2022). Biologic vs synthetic mesh for single-stage repair of contaminated ventral hernias: a randomized clinical trial. JAMA Surg.

[10] Maskal SM (2023). Mediumweight polypropylene mesh fractures after open retromuscular ventral hernia repair: incidence and associated risk factors. Surg Endosc.

[11] Warren JA, McGrath SP, Hale AL, Ewing JA, Carbonell AM, Cobb WS (2017). Patterns of recurrence and mechanisms of failure after open ventral hernia repair with mesh. Am Surg.

[12] Sajid MS, Kalra L, Parampalli U, Sains PS, Baig MK (2013). A systematic review and meta-analysis evaluating the effectiveness of lightweight mesh against heavyweight mesh in influencing the incidence of chronic groin pain following laparoscopic inguinal hernia repair. Am J Surg.

[13] Krpata DM (2021). Effect of hernia mesh weights on postoperative patient-related and clinical outcomes after open ventral hernia repair: a randomized clinical trial. JAMA Surg.

[14] Haskins IN (2018). A call for standardization of wound events reporting following ventral hernia repair. Hernia.

[15] Sadava EE, Krpata DM, Gao Y, Rosen MJ, Novitsky YW (2014). Wound healing process and mediators: implications for modulations for hernia repair and mesh integration: wound healing process and mediators. J Biomed Mater Res A.

[16] Andrades P, Prado A (2007). Composition of postabdominoplasty seroma. Aesthetic Plast Surg.

[17] Bakker WJ, Aufenacker TJ, Boschman JS, Burgmans JPJ (2021). Heavyweight mesh is superior to lightweight mesh in laparo-endoscopic inguinal hernia repair: a meta-analysis and trial sequential analysis of randomized controlled trials. Ann Surg.

[18] Jin J, Schomisch S, Rosen MJ (2009). In vitro evaluation of the permeability of prosthetic meshes as the possible cause of postoperative seroma formation. Surg Innov.

[19] Pochert N (2023). Th2/Th17 cell associated cytokines found in seroma fluids after breast cancer surgery. Arch Gynecol Obstet.

[20] Krpata DM (2017). Drain placement does not increase infectious complications after retromuscular ventral hernia repair with synthetic mesh: an AHSQC analysis. J Gastrointest Surg.

[21] Lu R (2020). Comparative review of outcomes: laparoscopic and robotic enhanced-view totally extraperitoneal (eTEP) access retrorectus repairs. Surg Endosc.

[22] Arora E (2022). Are drains useful in eTEP ventral hernia repairs? An AWR surgical collaborative (AWRSC) retrospective study. Surg Endosc.

[23] Miller BT (2022). Retromuscular drain versus no drain in robotic retromuscular ventral hernia repair: a propensity score-matched analysis of the abdominal core health quality collaborative. Hernia.

[24] Marcolin P (2023). Drain placement in retromuscular ventral hernia repair: a systematic review and meta-analysis. Hernia.

[25] Gurusamy KS, Allen VB (2013). Wound drains after incisional hernia repair. Cochrane Database Syst Rev.

[26] Kushner B, Smith E, Han B, Otegbeye E, Holden S, Blatnik J (2021). Early drain removal does not increase the rate of surgical site infections following an open transversus abdominis release. Hernia.

[27] Durai R, Mownah A, Philip CHN (2009). Use of drains in surgery: a review. J Perioper Pract.

